# Athlete anxiety questionnaire: the development and validation of a new questionnaire for assessing the anxiety, concentration and self-confidence of athletes

**DOI:** 10.3389/fpsyg.2023.1306188

**Published:** 2023-12-07

**Authors:** Melinda Trpkovici, Ágnes Pálvölgyi, Alexandra Makai, Viktória Prémusz, Pongrác Ács

**Affiliations:** ^1^Doctoral School of Health Sciences, Faculty of Health Sciences, University of Pécs, Pécs, Hungary; ^2^Institute of Physiotherapy and Sport Science, Faculty of Health Sciences, University of Pécs, Pécs, Hungary; ^3^Physical Activity Research Group, Szentágothai Research Centre, Pécs, Hungary

**Keywords:** athlete anxiety questionnaire, sports psychology, self-confidence, concentration, sports stress

## Abstract

**Introduction:**

Anxiety is one of the most prevalent issues among athletes. Therefore, measuring the stress caused by high-stakes situations could be important for investigating the issue. In sports psychology literature, no valid and reliable questionnaire is available in Hungarian for assessing the anxiety experienced by athletes in high-stakes situations. This study aimed to create a new Hungarian questionnaire to measure anxiety, self-confidence, and concentration during high-stakes contests.

**Methods:**

263 athletes of various sports participated in the cross-sectional study (age: 16.18 ± 3.46 years). The structure of the Anxiety Athletes Questionnaire (AAQ) was examined through factor analysis, where exploratory factor analysis (EFA) as well as confirmatory factor analysis (CFA) were carried out. The internal consistency of the subscales of AAQ was measured by Cronbach’s alpha. Through a convergent validity test, the AAQ questionnaire was compared to the subscales of the CSAI-2 and ACSI-28 questionnaire subscales by Spearman’s rank correlation coefficients. Through a discriminant validity analysis, the differences by age group, sex, and sport variables were examined by AAQ scores. The Mann–Whitney U and Kruskal-Wallis H tests were utilized in the analysis. The SPSS 28.0 software was used for the statistical analysis, and the level of significance was set at *p* < 0.05.

**Results:**

Four factors have been identified through the EFA. The CFA analysis showed the four-factor model an acceptable model fit (SRMR, RMSEA CFI, TLI). Cronbach’s alpha of the four subscales showed acceptable internal consistency (cognitive anxiety: α = 0.871; somatic anxiety: α = 0.700; self-confidence: α = 0.832; concentration: α = 0.747). The convergent validity showed a weak or moderate, significant relationship between AAQ subscales and subscales of CSAI and ACSI (R = −0.398–0.412).

**Conclusion:**

The Athlete Anxiety Questionnaire can be considered a reliable and valid measurement tool for measuring athletes’ anxiety, self-confidence and concentration in high-stakes situations.

## 1 Introduction

Regular physical activity has a beneficial effect on mental health. It can decrease psychological stress (Ács et al., 2016, 2020b; Jurak et al., 2020; WHO, 2022). By reducing anxiety and improving mood, sports support the maintenance of psychological health, but it can also be a stress factor that can cause anxiety (Ács et al., 2020a; Diotaiuti et al., 2021a). Anxiety is a variable that is widely investigated in sports psychology and has been the focal point of numerous studies (Jones, 1995; Mellalieu et al., 2009; Omar et al., 2014; Hardy et al., 2018; Diotaiuti et al., 2021b). Several reviews have been published to offer an interesting and informative insight into the relationship between competitive anxiety and performance (Jones, 1995; Smith et al., 1998; Whitehead and Duda, 1998; Gould et al., 2002; Fletcher and Fletcher, 2005; Woodman et al., 2010; Hardy et al., 2018; Tóth et al., 2022). Frequently, athletes experience performance-related concerns that lead to heightened anxiety, manifesting as muscle tension, sweaty palms, and the emergence of pessimistic thoughts regarding both the outcome of critical moments and their own capabilities (Crust and Azadi, 2010; Marín-González et al., 2022). Anxiety is an unpleasant, tense emotional state that is accompanied by a high activation of the autonomic nervous system and negative feelings and thoughts (Atkinson, 2005; Sangervo et al., 2022). Spielberger has made a distinction between two types of anxiety: trait anxiety and state anxiety (Spielberger, 1975).

State anxiety manifests itself only in specific situations (e.g., competitions), while trait anxiety shows the individual’s general anxiety tendency, i.e., it can be considered a personality trait. The different types of anxiety have been distinguished for a long time. A distinction can be made also between cognitive anxiety and somatic anxiety (Wine, 1971; Deffenbacher, 1977; Smith et al., 1998; Whitehead and Duda, 1998; Anshel and Wells, 2000; Gill et al., 2004; Mercader-Rubio et al., 2023). Cognitive anxiety manifests as lasting worrying, ruminating and dwelling over something, and it causes the deterioration of concentration, which affects performance and may lead to the likelihood of making mistakes (Morris et al., 1981; Lee et al., 2022). By contrast, somatic anxiety is the psychological and emotional aspect of the experience of anxiety and it is primarily associated with the direct activation of the autonomic nervous system (Liebert and Morris, 1967; Mercader-Rubio et al., 2023). Gould et al. (1987) believed that somatic anxiety directly affects the performance directly (Gould et al., 1987).

There are relatively few questionnaires for measuring anxiety within the field of sports psychology. The Sports Competition Anxiety Test (SCAT; Martens, 1977) is a multidimensional measurement tool that can be used to determine the anxiety levels of athletes; however, the test does not differentiate between somatic and cognitive anxiety and does not measure the differences between the two. The Sport Anxiety Scale (SAS; Smith et al., 1990) is a multidimensional anxiety measurement questionnaire that distinguishes between cognitive and somatic anxiety. This test can be used for measuring athletes’ trait anxiety. The questionnaire explores how the athlete usually feels before and during contests. The currently available valid Hungarian tests for measuring anxiety only measure the athlete’s state before the high-stakes contest, and the general mental state of the athlete (Sipos et al., 1999). As for sports performance, besides the level of anxiety, measuring the level of concentration is also necessary. Anxiety may cause deteriorated concentration, which is one of the most prominent precursors of negative performance (Smith et al., 1990). Furthermore, concentration can have correlations not only with the level of anxiety but with self-confidence as well. Athletes who have higher self-confidence have lower anxiety levels and are more able to focus on the task (Jones, 1995; Perry and Williams, 1998; Kaplánová, 2019; Ita et al., 2022). A review of the literature reveals that two types of anxiety, self-confidence and concentration, can both be associated with sports performance, which was the starting point for creating a questionnaire suitable for measuring all 4 factors.

Two questionnaires have served as the basis for the development of our questionnaire. The Competitive State Anxiety Inventory-2 (CSAI-2) questionnaire investigates competition anxiety, and self-confidence related to the result of the competition (Martens et al., 1990). It is based on the theory of Martens who has distinguished between three dimensions of anxiety (Martens, 1977). The first one is *cognitive anxiety,* which is the anticipation of failure and excessive worrying about its consequences. In this case, the athlete is worried about their performance and abilities.

*Somatic anxiety* is a tension that manifests in physical symptoms. The third factor is *self-confidence,* which refers to the level of the individual’s faith in themself and their abilities. The questionnaire was adapted to Hungarian by Sipos et al. (1999). The Athletic Coping Skills Inventory-28 (ACSI-28) questionnaire can be used for measuring athletes’ coping strategies (Smith et al., 1995). The Hungarian version of the test was created by Jelinek and Oláh and it provides an insight into athletes’ experience related to competitions and training sessions (Jelinek and Oláh, 2000). The measurement tools used in sports psychology have been developed for examining the states that precede the high-stakes situation.

The aim of this study was the development of a Hungarian sports psychology questionnaire that can be used for measuring with a multidimensional approach anxiety in athletes during high-stakes situations and for measuring the concentration and self-confidence of athletes, which are both essential factors in sports performance, and can have connections to anxiety.

## 2 Methods

### 2.1 Study design and participants

The cross-sectional quantitative study was conducted between March 2020–December 2022 in Pécs, Hungary. The determination of the sample size was based on the recommendation that a ratio of 10 participants per item is required for conducting factor analysis (MacCallum et al., 1999). The targeted sample size was 480 exploratory factor analyses for (EFA) and confirmatory factor analysis (CFA). Our study coincided with the first wave of the COVID-19 pandemic, and thus we were unfortunately faced with a significant drop in the number of the sample. 273 athletes of various sports aged 12–34 years were involved in the study, through convenience sampling. The final sample consisted of 263 athletes; 10 participants were excluded because of inadequately filled questionnaires. The mean age of the final sample was 16.18 ± 3.46 years. In terms of the gender ratio, 16.79% (44) were females and 83.21% (218) were males. All the participants or in the case of minors their parents were informed about the research and have provided written consent permitting the participation in the study. The data were processed anonymously. The Institutional Review Board of the Regional Research Committee of the Clinical Center, Pécs, Hungary granted permission for the study (No.:8821–5/2019/EÜIG). The research was performed in accordance with the Declaration of Helsinki.

#### 2.1.1 Inclusion criteria

To meet the *inclusion criteria*, individuals had to be certified athletes with a minimum of 1 year of sports club participation, engaging in training sessions at least three times per week for one and a half hours each. Additionally, participants needed to be at least 12 years old and were required to sign the informed consent form, or if underage, have their parent or guardian provide consent following a study briefing.

#### 2.1.2 Exclusion criteria

Receiving psychiatric treatment and taking medications were specified as *exclusion criteria*. No athlete had to be excluded due to the applied exclusion criteria.

### 2.2 Development process

The questionnaire is based on the Competitive State Anxiety Inventory-2 (CSAI-2; Martens et al., 1990) and the ACSI-28 (Athletic Coping Skills Inventory-28) questionnaires (Smith et al., 1995). During the pilot test, the questionnaire was tested among athletes with the participation of 20 people even before the data collection, then the final questionnaire was created based on the results of the expert group and the pilot study, and the psychometric properties of the instrument were examined. Based on the mentioned two questionnaires, the questions were formulated considering the purpose of the measurement. Based on the Hungarian version of the previous two questionnaires (Sipos et al., 1999; Jelinek and Oláh, 2000). Development of the Hungarian version of the questionnaire Athlete Anxiety Questionnaire was prepared using the validity criteria of the Delphi method and COSMIN checklist to manage the development process of the new measurement tool (Mokkink et al., 2010). The questionnaire was created with an expert group with the involvement of sports psychologists, sports coaches, and physical education teachers, and the statistical processing of the data was carried out by statisticians.

#### 2.2.1 Competitive state anxiety inventory-2

The measurement tool consists of 27 statements. The study subjects had to provide answers through a four-point Likert scale where 1 means “not at all” and 4 means “very much.” The *Cognitive anxiety* subscale shows worries related to the anticipated results of the performance, while the *Somatic anxiety* subscale measures physical symptoms triggered by stress.

The *Self-confidence* subscale related to the result of the competition aims to assess the extent to which the athlete has faith in a good result and in that their work invested will yield returns in a high-stakes situation. Higher scores mean more intense anxiety and a higher level of self-confidence related to competing (Martens et al., 1990).

#### 2.2.2 Athletic coping skills inventory-28

The questionnaire consists of 28 items, the items are grouped into 7 subscales, and each scale has 4 items, and the minimum score is 4 while the maximum score is 16. The scales of the questionnaire are the following: The *Coping with adversity* subscale measures the athlete’s behavior in unexpected and challenging situations that affect their performance. The *Peaking under pressure* scale measures performance in high-stakes situations and the extent to which the competitive situation, expectations and the need for approval influence the athlete’s performance. The *Goal setting and mental preparation* scale measures the objectives set by the athlete and their mental preparedness. *Concentration* measures the athlete’s level of concentration related to the competitive and training situation. The *Freedom from worry* scale measures the extent to which the athlete is filled with anxiety if their sports performance does not reach the required level, and about how others judge their performance. The *Confidence and achievement motivation* scale shows the level of confidence and positive motivation of the athlete. The *Coachability* scale aims to measure the relationship between the athlete and the coach (Smith et al., 1995).

### 2.3 Characteristics of the anxiety athletes questionnaire

The Anxiety Athletes Questionnaire (AAQ) is a new sports psychology measurement tool created by the authors that can be used for the measurement and interpretation of psychological anxiety. Furthermore, the questionnaire can also be used for the assessment of the level of concentration and self-confidence experienced during high-stakes contests. The final version of the measurement tool consists of 20 items that are grouped into 4 factors. Cognitive anxiety is the anticipation of failure and excessive worrying about its consequences (e.g.: 1. *“During the match/contest, I was worried that I would not perform the way I would have liked to.”*). Somatic anxiety is the tension that manifests in physical symptoms (e.g.: 8.) (*“During the match/contest, my stomach felt tight.”*). Self-confidence refers to the level of the individual’s faith in themself and their abilities (e.g.: 10. *“During the match/contest, my self-confidence helped me play well.”*). The Concentration scale measures the athlete’s level of concentration related to the competitive situation (e.g.: 13. *“During the match/contest, I was able to focus on one thing or person.”*). The scales and their respective items are the following:

1. C*ognitive Anxiety* items: 1,5,9,11,14,18.
2. *Somatic Anxiety* items: 4,8,17.
3. *Self-confidence* items: 2,6,10,12,15,19.
4. The *Concentration* items: 3,7,13,16,20 (from which reverse items: 3,7,20).

The questionnaire asks questions about feelings, emotions and thoughts triggered by the actual stress experienced. The test includes statements that are habitually used by athletes to describe their states during competition. The study subjects had to provide answers through a four-point Likert scale where 1 means “not at all” and 4 means “very much.” These abovementioned factors are the subscales of the questionnaire. The total scores are calculated by adding up the scores given for each item. The maximum scores for individual scales are the following: *Cognitive anxiety:* 24 points, *Somatic anxiety:* 12 points, *Self-confidence:* 24 points, and *Concentration:* 20 points.

#### 2.3.1 Data collection procedure

The authors distributed the questionnaires to the coaches, who asked the athletes on the day of the match to complete them. Following the review and endorsement of the study information sheet and consent form, athletes completed the demographic data sheet, as well as the ACSI-28 and CSAI-2 questionnaires before the competition. This took approximately 20–25 min, after which they filled in the 3rd questionnaire (AAQ) after the competition, which took approximately 10–15 min. Participation in the study was voluntary and anonymous. The completed tests were returned to the authors by the coaches.

### 2.4 Statistical analysis

The newly developed questionnaire’s construct validity was evaluated through both exploratory factor analysis (EFA) and confirmatory factor analysis (CFA). In the EFA phase, we employed the Statistical Package for Social Sciences version 27 (SPSS, Chicago, IL, United States). To verify the appropriateness of the data for EFA, the Kaiser-Meyer-Olkin (KMO) measure and Bartlett’s test of sphericity were employed. Principal components analysis was utilized to extract factors.

To calculate CFA, we used IBM AMOS 29.0 (SPSS Chicago, IL, United States) software. Given the sensitivity of Chi-square statistics and their associated *p*-values to sample size, this study utilized the Chi-square test divided by its degrees of freedom as a consideration.

Comparative fit index (CFI > 0.95 good fit) and goodness of fit index (GFI > 0.95 good fit) were reported as incremental fit statistics and the root-mean-square error of approximation (RMSEA <0.08 good fit) Tucker- Lewis index (TLI > 0.95 good fit) was calculated (Brown, 2015) as well.

To evaluate internal consistency and reliability, we employed Cronbach’s alpha coefficient, with a threshold of ≥0.70 being deemed acceptable (Taber, 2018). The concurrent validity was assessed by Spearman’s rank correlation coefficient (R) for correlations between the measured scales (Carlson and Herdman, 2012).

The statistical analyses were conducted using the SPSS 27.0. software, where values *p* ≤ 0.05 were considered significant.

## 3 Results

In the study 273 athletes of various sports aged 12–34 years were involved, through convenience sampling. The final sample consisted of 263 athletes; 10 participants were excluded because of inadequately filled questionnaires. The mean age of the final sample was 16.18 ± 3.46 years. In terms of the gender ratio, 16.79% (44) were females and 83.21% (218) were males. The largest proportion of participants in the sample were volleyball players (35.9%); this was followed by football players (32.7%), then water polo players (20.9%), basketball players (6.5%) and finally table tennis players (3.9%).

The structure of the questionnaire was examined through factor analysis with the execution of exploratory factor analysis (EFA) and confirmatory factor analysis (CFA). Through the EFA (*N* = 106), we first assessed the suitability of the data for factor analysis. According to Bartlett’s test (*p* < 0.001) and the Kaiser-Meyer-Olkin (KMO) criterion (0.818), the data were suitable for EFA.

The factor structure of the AAQ questionnaire was carried out through principal component analysis. The distribution of variance among the 20 items across four factors accounted for a total of 63.34%. Specifically, the cognitive factor contributed to 24.83% of the variance, self-confidence explained 15.49%, concentration accounted for 11.05%, and the somatic factor elucidated 11.86%. The rotated factor matrix (varimax rotation) showed the following structure. See correlation coefficients with an absolute value higher than 0.300 were included in Table 1.

**Table 1:** The factor structure of the anxiety athletes questionnaire (*N* = 106).

	Component			
	Cognitive anxiety	Self-confidence	Concentration	Somatic anxiety
Item 1	0.842			
Item 2		0.425		
Item 3			0.580	
Item 4				0.679
Item 5	0.800			
Item 6		0.639		
Item 7			0.544	
Item 8				0.550
Item 9	0.493			
Item 10		0.734		
Item 11	0.819			
Item 12		0.817		
Item 13			0.523	
Item 14	0.843			
Item 15		0.649		
Item 16			0.545	
Item 17				0.522
Item 18	0.535			
Item 19		0.620		
Item 20			0.572	

Our findings have also been supported by confirmatory factor analysis (*N* = 157), carried out with the IBM AMOS 29.0 software. The goodness of the model was assessed based on several fit indexes. One of the most frequently used fit indexes is the Chi-square test. The test resulted in chi-square = 201.414, *df* = 154, *p* = 0.056, chi-square/df = 1.308 in our CFA. The value of the Chi-square is considered adequate as it is lower than double of the degree of freedom, and the value of p is higher than 0.05. For the assessment of the goodness of fit, we have utilized the comparative fit index (CFI = 0.960) and the Tucker-Lewis index (TLI = 0.951), and the root mean square error of approximation (RMSEA = 0.044) value. The CFI and TLI indexes have shown an acceptable, close fit. According to the RMSEA value of 0.044 (0.049–0.076), the fit of the data is appropriate.

### 3.1 Internal consistency test

In our study, the internal consistency of the various questions of the subscales is provided by the correlation between the questions, indicated by Cronbach’s alpha. The relevant values of the four subscales are summarized in Table 2.

**Table 2:** The internal consistencies of the subscales of the anxiety athletes questionnaire.

	Cognitive anxiety	Self-confidence	Concentration	Somatic anxiety
Cronbach alpha	0.871	0.832	0.747	0.700

### 3.2 Convergent validity

Through a convergent validity test, the AAQ questionnaire was compared to the subscales of the CSAI-2 and ACSI-28 questionnaires. For the analysis of the correlations between the measurement tools, Spearman’s rank correlation analysis was used, the results are summarized in Table 3.

**Table 3:** Correlations between the competitive state anxiety inventory-2, athletic coping skills inventory-28 and anxiety athletes questionnaire (*N* = 263).

			AAQ			
			Somatic anxiety	Cognitive anxiety	Self-confidence	Concentration
ACSI-28	Coping with adversity	R	−0.143*	−0.316**	0.412**	0.266**
		p	0.021	0.000	0.000	0.000
	Peaking under pressure	R	−0.212**	−0.071	0.414**	0.191**
		p	0.001	0.253	0.000	0.002
	Goal setting and mental preparation	R	−0.122*	−0.042	0.357**	0.178**
		p	0.049	0.497	0.000	0.004
	Concentration	R	−0.218^**^	−0.277^**^	0.447^**^	0.280^**^
		p	0.000	0.000	0.000	0.000
	Freedom from worry	R	−0.253**	−0.408**	0.321**	0.214*
		p	0.000	0.000	0.000	0.001
	Confidence and achievement motivation	R	−0.146*	−0.155*	0.440**	0.194**
		p	0.019	0.012	0.000	0.002
	Coachability	R	−0.107	−0.176**	0.221**	0.154*
		p	0.085	0.004	0.000	0.013
CSAI-2	Cognitive anxiety	R	0.210**	0.411**	−0.257**	−0.251**
		p	0.001	0.000	0.000	0.000
	Somatic anxiety	R	0.057	0.564**	−0.308**	−0.398**
		p	0.357	0.000	0.000	0.000
	Self-confidence	R	−0.219**	−0.372**	0.654**	0.389**
		p	0.000	0.000	0.000	0.000

CSAI-2, Competitive State Anxiety Inventory-2; ACSI-28, Athletic Coping Skills Inventory-28; AAQ, Anxiety Athletes Questionnaire *p ≤ 0.05, **p ≤ 0.01.

### 3.3 Discriminant validity

Based on the Mann–Whitney U-test, a significant difference was found in cognitive, self-confidence and concentration subscales between genders (*p* < 0.05). The level of cognitive anxiety was higher in female athletes (11.67 ± 3.97 vs. 9.26 ± 3.23), but in self-confidence (19.54 ± 3.08 vs. 17.47 ± 3.74) and concentration (18.07 ± 1.86 vs. 17.73 ± 1.96), in males scored significantly higher. The somatic subscale values were almost identical, the difference was not significant (*p* = 0.756; Table 4).

**Table 4:** Discriminant validity of the subscales of the anxiety athletes questionnaire (*N* = 263), gender differences based on Mann-Witney U-test.

		Somatic anxiety	Cognitive anxiety	Self-confidence	Concentration
Male	Mean	3.34	9.26	19.54	18.07
	SD	0.85	3.23	3.08	1.86
Female	Mean	3.38	11.67	17.47	17.73
	SD	0.81	3.97	3.74	1.96
Total	Mean	3.35	9.95	18.95	17.97
	SD	0.84	3.62	3.41	1.89
Gender difference	Z	−0.311	−3.760	−3.247	−1.049
	p	0.756	<0.001	0.001	0.294

## 4 Discussion

The goal of this study was to develop and validate a new sports psychology questionnaire, that can be used for measuring anxiety, concentration, and self-confidence during high-stakes situations. The measurement tools of anxiety in athletes currently available in Hungarian can only be used for assessing athletes’ states before the high-stakes situation. The development of the new measurement tool was based on questionnaires that are used for measuring anxiety in athletes and that have a valid Hungarian version. The structure of the questionnaire was examined through factor analysis, where the EFA was complemented by a CFA. These have shown that the variables were suitable for conducting an EFA. The factor structure of the AAQ questionnaire was carried out through principal component analysis. The variance explained by the 4 factors of 20 items was 63.34%. The items of the questionnaire were grouped into *cognitive anxiety, somatic anxiety, concentration,* and *self-confidence* factors.

Our findings have also been supported by a confirmatory factor analysis. The goodness of the model was assessed based on several fit indexes. The new questionnaire has been tested with the participation of athletes. The study’s key findings indicate the successful development of a valid and reliable questionnaire, confirming the validity of the three-factor anxiety model proposed by Martens et al. (1990). The components of cognitive anxiety are present in the individual’s thoughts about their own abilities, influenced by the fear of potential failure.

Somatic anxiety constitutes the physiological effects of the experience itself: increased arousal levels accompanied by negative physical symptoms. The self-confidence shows the level of confidence associated with competition (Woodman et al., 2010; Gyömbér, 2018; Ita et al., 2022). Studies about competition anxiety have shown that a more intense level of fear was triggered in athletes whose competition-related anxiety levels were higher, compared to those whose anxiety levels were lower (Hajdúné László, 2009; Ita et al., 2022). Consequently, measuring anxiety in athletes is particularly important. The better we are able to study anxiety the more effectively the coaching of athletes can be organized. The ability to manage one’s anxiety in a stressful situation is important for successful performance (Smith et al., 1995; Géczi et al., 2008; Tóth et al., 2022). Teaching anxiety reduction methods, confidence building, and concentration development, which all contribute to performance enhancement, are pivotal parts of the sports psychology work process (Ágota, 2002; Krisztina, 2013; Fadare et al., 2022).

In the literature, there are anxiety tests that measure cognitive and somatic anxiety. The Sport Anxiety Scale-2 (SAS-2) is a questionnaire suitable for measuring anxiety in athletes before or during competition (Smith et al., 2006). From the value of anxiety measured with the SAS-2 scale, a constant tendency can be inferred, which indicates the general experience of the athlete in the situations before and during the competition. In contrast, state anxiety is a psychological and physiological reaction experienced during a specific competition, and does not appear as a permanent feature. When measuring anxiety as a trait, athletes are asked about the general reaction they experience during the competition, while when measuring anxiety as a state, the answers refer to a specific situation (Leal et al., 2017; Mercader-Rubio et al., 2023). Looking at gender differences, women can be characterized by a higher level of competitive anxiety than men. This relationship concerned particularly women practicing sport recreationally (Tomczak et al., 2022). In our study, we also found significant gender differences in cognitive, self-confidence and concentration subscales.

The Sport Competition Anxiety Test (SCAT) is also among the tests that measure competitive anxiety (Martens, 1977). The questions of the SCAT are aimed at how the athlete feels in a competitive situation. The degree of anxiety can be deduced from the total score of the SCAT. Individual anxiety levels affect group performance and team results. Sopa’s study emphasizes that early detection of errors in teamwork and individual anxiety levels can lead to better team performance and improved levels of cohesion and communication (Sopa and Pomohaci, 2020).

Our questionnaire was supplemented with the self-confidence factor, which shows a close correlation with anxiety (Gyömbér, 2018; Ita et al., 2022). Furthermore, supplemented with a concentration factor, which is also related to anxiety level and self-esteem. Athletes with higher self-esteem, have lower anxiety levels and better ability to focus on the task (Kaplánová, 2019; Ita et al., 2022). It seems to be a suitable tool with regard to the cognitive and somatic aspects of anxiety not only for basic research but also for the assessment of the effectiveness of interventions aimed at reducing anxiety. Furthermore, AAQ may be useful for customizing interventions for individual athletes with differing somatic and cognitive anxiety patterns. For example, interventions aimed at arousal regulation, such as relaxation training, may be particularly useful for athletes with high levels of somatic anxiety, while athletes with high levels of cognitive anxiety or concentration issues may benefit from more cognitively oriented interventions. Irrational beliefs are positively related to cognitive and somatic competitive anxiety in athletes (Chadha et al., 2019). The findings of Tóth’s study show that athletes with more rigid, extreme and illogical thinking (irrational beliefs) are more worried about their performance and possible negative consequences (cognitive anxiety). This may be due to their excessive expectations, as well as a critical attitude toward their performance (Tóth et al., 2022). Several interventional tools have been shown to be effective in reducing cognitive trait anxiety, such as the reframing technique. With the help of this method, the athlete can transform the negative content in his mind into positive content. In this case, negative content from the point of view of sports performance becomes content that helps positive sports performance (Kaplánová, 2020). We recommend the use of various sports psychology interventions for the development of athletes, for example the use of cognitive techniques, e.g., reframing to reduce anxiety about cognitive traits.

## 5 Limitations

The study was not randomized. It would be useful to conduct our research with a larger sample, however, several contests were canceled due to the COVID-19 pandemic or access to them was limited, and thus our access to testing athletes was significantly reduced. Our goal is to examine our research with a larger number of sample elements, as well as to expand the research according to the types of sports which also makes it suitable for measuring the differences between sports.

## 6 Conclusion

The Athlete Anxiety Questionnaire can be considered a reliable and valid measurement tool for measuring athletes’ anxiety, self-confidence and concentration in high-stakes situations. All four factors can be linked to sports performance, which is why we emphasize the importance of mental preparation. During the interventions, it is important that the athletes acquire techniques that can effectively manage stress, improve their concentration skills, and improve their self-confidence.

The novelty of our questionnaire lies in the fact that it asks about the experience of the high-stakes situation, in contrast to the questionnaires found so far, which deal with the situation before the high-stakes situation. Furthermore, in addition to maintaining the three-factor anxiety model, the concentration factor was added, so we can expand the measurement of effectiveness with an additional factor within a questionnaire, which can form the basis of future research.

## Data availability statement

The raw data supporting the conclusions of this article will be made available by the authors, without undue reservation.

## Ethics statement

The studies involving humans were approved by Institutional Review Board of the Regional Research Committee of the Clinical Center, 7,622 Pécs, Hungary, Vasvári str. 4. The studies were conducted in accordance with the local legislation and institutional requirements. Written informed consent for participation in this study was provided by the participants’ legal guardians/next of kin.

## Author contributions

MT: Conceptualization, Data curation, Investigation, Validation, Writing – original draft, Writing – review & editing. AP: Conceptualization, Writing – review & editing. AM: Formal analysis, Methodology, Software, Validation, Visualization, Writing – original draft, Writing – review & editing. VP: Conceptualization, Formal analysis, Supervision, Writing – review & editing. PÁ: Conceptualization, Funding acquisition, Resources, Supervision, Writing – review & editing.
